# Air Pollution Exposure During Pregnancy, Ultrasound Measures of Fetal Growth, and Adverse Birth Outcomes: A Prospective Cohort Study

**DOI:** 10.1289/ehp.1003316

**Published:** 2011-08-26

**Authors:** Edith H. van den Hooven, Frank H. Pierik, Yvonne de Kluizenaar, Sten P. Willemsen, Albert Hofman, Sjoerd W. van Ratingen, Peter Y.J. Zandveld, Johan P. Mackenbach, Eric A.P. Steegers, Henk M.E. Miedema, Vincent W.V. Jaddoe

**Affiliations:** 1 The Generation R Study Group, Erasmus Medical Center, Rotterdam, the Netherlands; 2 Urban Environment and Safety, TNO, Utrecht, the Netherlands; 3 Department of Epidemiology,; 4 Department of Biostatistics,; 5 Department of Public Health,; 6 Department of Obstetrics and Gynaecology, and; 7 Department of Paediatrics, Erasmus Medical Center, Rotterdam, the Netherlands

**Keywords:** air pollution, birth weight, dispersion modeling, fetal growth, intrauterine growth restriction, nitrogen dioxide, particulate matter, pregnancy, preterm birth

## Abstract

Background: Air pollution exposure during pregnancy might have trimester-specific effects on fetal growth.

Objective: We prospectively evaluated the associations of maternal air pollution exposure with fetal growth characteristics and adverse birth outcomes in 7,772 subjects in the Netherlands.

Methods: Particulate matter with an aerodynamic diameter < 10 μm (PM_10_) and nitrogen dioxide (NO_2_) levels were estimated using dispersion modeling at the home address. Fetal head circumference, length, and weight were estimated in each trimester by ultrasound. Information on birth outcomes was obtained from medical records.

Results: In cross-sectional analyses, NO_2_ levels were inversely associated with fetal femur length in the second and third trimester, and PM_10_ and NO_2_ levels both were associated with smaller fetal head circumference in the third trimester [–0.18 mm, 95% confidence interval (CI): –0.24, –0.12 mm; and –0.12 mm, 95% CI: –0.17, –0.06 mm per 1-μg/m^3^ increase in PM_10_ and NO_2_, respectively]. Average PM_10_ and NO_2_ levels during pregnancy were not associated with head circumference and length at birth or neonatally, but were inversely associated with birth weight (–3.6 g, 95% CI: –6.7, –0.4 g; and –3.4 g, 95% CI: –6.2, –0.6 g, respectively). Longitudinal analyses showed similar patterns for head circumference and weight, but no associations with length. The third and fourth quartiles of PM_10_ exposure were associated with preterm birth [odds ratio (OR) = 1.40, 95% CI: 1.03, 1.89; and OR = 1.32; 95% CI: 0.96, 1.79, relative to the first quartile]. The third quartile of PM_10_ exposure, but not the fourth, was associated with small size for gestational age at birth (SGA) (OR = 1.38; 95% CI: 1.00, 1.90). No consistent associations were observed for NO_2_ levels and adverse birth outcomes.

Conclusions: Results suggest that maternal air pollution exposure is inversely associated with fetal growth during the second and third trimester and with weight at birth. PM_10_ exposure was positively associated with preterm birth and SGA.

Maternal exposure to air pollution during pregnancy has been suggested to be associated with increased risks of adverse birth outcomes such as low birth weight, intrauterine growth restriction, and preterm birth (Ritz and Wilhelm 2008). Thus far, results are not consistent: Reported associations (or absence thereof) for specific air pollutants, exposure periods, and birth outcomes have differed between studies (Bonzini et al. 2010; Shah and Balkhair et al. 2011). Most previous studies defined fetal growth using measures at birth, such as weight, length, and head circumference (Choi et al. 2006; Hansen et al. 2007; Jedrychowski et al. 2004; Salam et al. 2005). However, because impaired growth during early pregnancy may be compensated for in the remaining intrauterine life, the eventual measures at birth can represent both normal and abnormal fetal growth and development. To provide insight into the specific effects of maternal air pollution exposure and to identify critical windows of exposure, it is of interest to assess fetal growth in different periods of pregnancy rather than only at birth. A small number of studies have examined the impact of air pollution exposure on fetal growth using ultrasound measurements during pregnancy as direct estimates of growth (Aguilera et al. 2010; Hansen et al. 2008; Slama et al. 2009). These studies were based on small numbers, did not report measurements in each trimester of pregnancy, or were not able to consider the spatiotemporal variation in air pollution exposure.

We investigated associations of particulate matter with an aerodynamic diameter < 10 μm (PM_10_) and nitrogen dioxide (NO_2_) exposure levels during pregnancy with fetal growth characteristics assessed by ultrasound in each trimester of pregnancy and adverse birth outcomes in a population-based cohort study among 7,772 pregnant women in the Netherlands.

## Methods

*Design.* This study was embedded in the Generation R Study, a population-based prospective cohort study from pregnancy onward in Rotterdam, the Netherlands (Jaddoe et al. 2010). Mothers were enrolled between 2001 and 2005. The study protocol was approved by the Medical Ethical Committee of Erasmus Medical Center, Rotterdam. Written consent was obtained from all participants. Of the 8,880 prenatally enrolled women, air pollution exposure estimates were available for 7,870 mothers (89%). For 1,010 mothers, air pollution concentrations could not be estimated because of incomplete address history or because they had moved outside the study area before delivery. Women with a twin pregnancy (*n* = 79), abortion (*n* = 7), or intrauterine death (*n* = 12) were excluded. The cohort for analysis consisted of 7,772 mothers and singleton live births [see Supplemental Material, Figure S1 (http://dx.doi.org/10.1289/ehp.1003316)].

*Air pollution exposure.* Individual exposures to PM_10_ and NO_2_ during pregnancy were assessed at the home address, using a combination of continuous monitoring data and dispersion modeling techniques, taking into account both the spatial and temporal variation in air pollution. A detailed description and flow chart of the exposure assessment are presented in Supplemental Material, pp. 3–6 and Figure S2 (http://dx.doi.org/10.1289/ehp.1003316). In brief, annual average concentrations of PM_10_ and NO_2_ for 2001–2006 were assessed for all addresses in the study area, using the three Dutch national standard methods for air quality modeling (Netherlands Ministry of Infrastructure and the Environment 2007). Subsequently, hourly concentrations of PM_10_ and NO_2_ were derived using hourly air pollution measurements from three continuous monitoring stations (hourly calibration), taking into account hourly wind conditions and fixed temporal patterns in source contributions. We obtained full residential history of the participants. Based on the home addresses of the participants, we derived individual exposure estimates for different periods in pregnancy: conception until first-trimester ultrasound, conception until second-trimester ultrasound, conception until early third-trimester ultrasound, and conception until delivery.

*Fetal growth characteristics.* Fetal ultrasound examinations were performed in each trimester of pregnancy. In the first trimester, we used fetal crown–rump length to assess fetal growth only in mothers with a known date of last menstrual period (LMP) and a regular menstrual cycle of 28 (range, 24–32) days (Mook-Kanamori et al. 2010). Date of LMP was obtained from the community midwife or hospital and was confirmed orally with the subjects at the ultrasound visit. For growth measurements in the second and third trimester we used gestational age based on ultrasound examination (Verburg et al. 2008b), because using LMP has several limitations (Tunon et al. 1996). Fetal growth measurements used in the present study included head circumference and femur length. Femur length was used as a proxy for fetal body length. Estimated fetal weight was calculated using femur length and head and abdominal circumference using the Hadlock formula (Hadlock et al. 1985). Longitudinal growth curves and gestational age adjusted standard deviation (SD) scores were constructed for all fetal growth measurements based on reference growth curves of our own study population (Verburg et al. 2008b).

*Birth outcomes.* Medical records and hospital registries were used to obtain information about date of birth, gestational age at birth, fetal sex, birth weight, and birth length. We completed missing data on birth length (16%) with measurements of length from the first visit at the routine child health center within the first 2 months after birth, which had negligible influence on the results. Head circumference was not routinely measured at birth; therefore, we used head circumference from the first child health center visit. The regression models with neonatal length or head circumference as the outcome were adjusted for postconceptional age (gestational age for measurements at birth or gestational age plus postnatal age for measurements at the child health center). Models with neonatal length were also adjusted for the method of measurement (birth or child health center). Gestational age and sex-adjusted SD scores for birth weight and birth length were constructed based on reference charts from a North European birth cohort (Niklasson et al. 1991). Postnatal age and sex-adjusted SD scores for neonatal head circumference and length were constructed using reference charts from a nationwide study in the Netherlands (Fredriks et al. 2000). The regression models with SD scores for neonatal head circumference or length were adjusted for gestational age at birth, and models with SD scores for neonatal length were also adjusted for the method of measurement. Adverse birth outcomes were defined as preterm birth (gestational age < 37 weeks), low birth weight (birth weight < 2,500 g), and small size for gestational age at birth (SGA) (gestational age and sex-adjusted birth weight less than fifth percentile).

*Covariates.* Information on maternal age, educational level (no education/primary, secondary, or higher), parity (nulliparous, multiparous), folic acid supplementation use (preconceptional, first 10 weeks of pregnancy, none) (Timmermans et al. 2009), and ethnicity was obtained by a questionnaire at enrollment. Because there were no differences in observed results when ethnicity was categorized by five groups instead of two, we classified ethnicity into European and non-European groups. Maternal smoking and alcohol consumption before and during pregnancy (no, yes) were assessed by questionnaires in each trimester. Maternal and paternal anthropometrics were assessed at enrollment. Road traffic noise exposure (*L_den_*) at the home address was assessed according to requirements of the European Environmental Noise Directive (European Commission 2002). The assessment procedure is described in more detail in Supplemental Material, pp. 8–9 (http://dx.doi.org/10.1289/ehp.1003316).

*Statistical analysis.* We used the lowest quartiles of PM_10_ and NO_2_ exposure as the reference exposure groups. First, with multivariate linear regression models, we assessed associations between air pollution exposure in quartiles in the relevant time periods (i.e., from conception until measurement) with absolute measures of fetal growth and neonatal parameters. Second, to assess potential nonlinear longitudinal effects, we used mixed-effects models with unstructured residual covariance to longitudinally model fetal growth SD scores from 18 weeks of pregnancy until birth by natural cubic splines (Devlin and Weeks 1986). We present these results as change in SD score to enable comparison of effect estimates throughout pregnancy. We positioned interior knots of the spline based on moments of data collection (18, 23, 30, 37, and 43.4 weeks for head circumference and weight and 10.5, 15, 25, 37, and 43.4 weeks for length). The models include a separate spline model for each quartile of air pollution exposure during pregnancy. We performed a multivariate *F*-test to test for a difference between the splines of each quartile of air pollution exposure compared with the reference group. Third, we assessed the associations between air pollution exposure during pregnancy and adverse birth outcomes using multivariate logistic regression analyses. Tests for trend were performed by including PM_10_ and NO_2_ exposure as a continuous variable in the linear or logistic regression models. All models were adjusted for known determinants of fetal growth (maternal age, body mass index, height, ethnicity, education, parity, folic acid supplementation use, smoking, alcohol consumption, paternal height) and for road traffic noise exposure. Models of absolute measures of fetal growth, fetal growth SD scores, preterm birth, and low birth weight were additionally adjusted for fetal sex. Models of absolute measures of fetal growth were additionally adjusted for gestational age at measurement. Models of low birth weight were additionally adjusted for gestational age at birth. The percentages of missing values within the population for analysis were < 1% for continuous data and < 15% for the categorical data, except for folic acid supplementation use (26%). We used multiple imputation to impute missing values for covariates (van Buuren 2007). All measures of association are presented with their 95% confidence intervals (CIs). Spline regression analyses were performed using SAS version 9.2 (SAS Institute Inc., Cary, NC, USA), and other analyses were performed using PASW version 17.0 for Windows (PASW Inc., Chicago, IL, USA).

## Results

*Subject and exposure characteristics.* Table 1 presents the maternal, paternal, and fetal characteristics. Data on air pollution exposure levels are presented in Table 2. Mean total exposure levels during pregnancy were 30.3 μg/m^3^ for PM_10_ and 39.8 μg/m^3^ for NO_2_. Correlations among exposure averages in different pregnancy periods were moderate to strong (PM_10_: Pearson correlation coefficient *r* = 0.76–0.96; NO_2_: *r* = 0.68–0.94). PM_10_ and NO_2_ exposure averages for corresponding periods were moderately correlated (*r* = 0.57–0.63). Figure S3 in Supplemental Material (http://dx.doi.org/10.1289/ehp.1003316) presents maps of the distribution of PM_10_ and NO_2_ concentrations in the study area, demonstrating substantial spatial differences in annual average concentrations (up to 4–8 μg/m^3^) between urban and suburban areas.

**Table 1:** Subject characteristics (*n* = 7,772).

	
	
	
	
Values are means ± SDs, medians (95% range) for variables with a skewed distribution, or number of subjects (percentage) for categorical variables.	

**Table 2:** Distribution of PM_10_ and NO_2_ exposure levels for different pregnancy periods.

Air pollution level	Minimum	25th percentile	Mean	Median	75th percentile	Maximum
PM_10_ exposure (μg/m^3^)												
Until first trimester		21.7		27.4		30.8		30.8		33.6		44.0
Until second trimester		22.6		28.0		30.7		30.6		33.6		43.2
Until third trimester		22.7		27.8		30.4		30.5		33.2		41.5
Total pregnancy		23.2		27.8		30.3		30.0		32.9		40.9
NO_2_ exposure (μg/m^3^)												
Until first trimester		21.0		37.0		40.4		40.9		43.9		59.7
Until second trimester		22.7		37.0		40.2		40.5		43.4		58.4
Until third trimester		25.6		37.0		40.0		39.8		42.8		58.2
Total pregnancy		26.5		37.2		39.8		39.6		42.2		56.9
Air pollution exposure was estimated for different periods in pregnancy: conception until first, second, and early third trimester ultrasound, and conception until delivery.												

*Air pollution and fetal growth characteristics.* Tables 3 and 4 present the cross-sectional associations for air pollution exposure with fetal growth characteristics. PM_10_ and NO_2_ levels were not consistently associated with second-trimester or neonatal head circumference, but higher levels were associated with smaller fetal head circumference in the third trimester (difference –0.18 mm, 95% CI: –0.24, –0.12 mm per 1-μg/m^3^ increase in PM_10_; and –0.12 mm, 95% CI: –0.17, –0.06 mm per 1-μg/m^3^ increase in NO_2_; *p*-values < 0.01). PM_10_ levels were not associated with fetal or neonatal length, but NO_2_ levels were inversely associated with fetal femur length in the second and third trimester (*p* < 0.01). Exposure to PM_10_ was associated with increased estimated fetal weight in the second trimester (*p* < 0.05), but PM_10_ and NO_2_ levels were associated with a lower birth weight (difference –3.6 g, 95% CI: –6.7, –0.4 g per 1-μg/m^3^ increase in PM_10_; and –3.4 g, 95% CI: –6.2, –0.6 g per 1-μg/m^3^ increase in NO_2_; *p*-values < 0.05).

**Table 3:** Trimester-specific associations of PM_10_ exposure with measures of fetal growth.

PM_10_ [difference (95% CI)]					Trend test (per 1-µg/m^3^ increase)	*p*-Value for trend
Fetal growth parameter	*n*	2nd quartile	3rd quartile	4th quartile		
Head circumference												
Second trimester (mm)		6,625		0.65 (0.24, 1.05)*		0.57 (0.16, 0.98)*		0.16 (–0.26, 0.57)		0.01 (–0.03, 0.05)		0.51
Third trimester (mm)		6,723		0.35 (–0.25, 0.94)		–0.43 (–1.02, 0.16)		–1.74 (–2.34, –1.13)**		–0.18 (–0.24, –0.12)		< 0.01
Birth (cm)		4,448		–0.01 (–0.09, 0.07)		–0.05 (–0.14, 0.04)		–0.03 (–0.13, 0.06)		0.00 (–0.02, 0.01)		0.39
Length												
First trimester (mm)		1,541		0.42 (–0.58, 1.41)		–0.35 (–1.34, 0.65)		–0.77 (–1.82, 0.28)		–0.08 (–0.17, 0.00)		0.06
Second trimester (mm)		6,646		0.14 (0.02, 0.27)*		–0.04 (–0.16, 0.08)		0.00 (–0.12, 0.12)		–0.01 (–0.02, 0.00)		0.15
Third trimester (mm)		6,778		0.11 (–0.04, 0.26)		0.00 (–0.15, 0.15)		–0.15 (–0.30, 0.00)*		–0.01 (–0.03, 0.00)		0.11
Birth (cm)		5,606		–0.07 (–0.21, 0.07)		–0.12 (–0.27, 0.02)		0.15 (–0.01, 0.30)^#^		0.02 (0.00, 0.03)		0.09
Weight												
Second trimester (g)		6,612		5.9 (2.9, 8.8)*		5.0 (2.1, 8.0)*		3.8 (0.8, 6.8)*		0.3 (0.0, 0.6)		0.05
Third trimester (g)		6,751		11.0 (–1.0, 22.9)^#^		7.4 (–4.7, 19.4)		–11.0 (–23.2, 1.2)^#^		–0.7 (–1.9, 0.6)		0.29
Birth (g)		7,003		–18.1 (–45.2, 9.1)		–25.5 (–52.8, 1.8)^#^		–34.3 (–62.1, –6.4)*		–3.6 (–6.7, –0.4)		0.03
Values are regression coefficients and reflect the difference in fetal growth parameters for each quartile of PM_10_ exposure (averaged from conception until measurement) compared with the reference group (lowest quartile). Cutoff values for categorization of PM_10_ exposure are < 27.4, 27.4–30.8, 30.8–33.6, > 33.6 μg/m^3^ for first trimester, < 28.0, 28.0–30.6, 30.6–33.6, > 33.6 μg/m^3^ for second trimester, < 27.8, 27.8–30.5, 30.5–33.2, > 33.2 μg/m^3^ for third trimester, and < 27.8, 27.8–30.0, 30.0–32.9, > 32.9 μg/m^3^ for total pregnancy. Models are adjusted for gestational age and noise exposure at measurement, maternal age, body mass index, height, parity, ethnicity, education, folic acid supplementation use, smoking, alcohol consumption, paternal height, and fetal sex. Models with neonatal head circumference or length are additionally adjusted for postconceptional age (gestational age for measurements at birth or gestational age plus postnatal age for measurements at the child health center), and models with neonatal length are additionally adjusted for method of measurement. *p < 0.05. **p < 0.001. ^#^*p* < 0.10.												

**Table 4:** Trimester-specific associations of NO_2_ exposure with measures of fetal growth.

NO_2_ [difference (95% CI)]							Trend test (per 1-µg/m^3^ increase)	*p*-Value for trend
Fetal growth parameter	*n*	2nd quartile	3rd quartile	4th quartile				
Head circumference												
Second trimester (mm)		6,625		0.16 (–0.25, 0.57)		–0.24 (–0.66, 0.17)		–0.23 (–0.69, 0.22)		–0.02 (–0.05, 0.02)		0.36
Third trimester (mm)		6,723		–0.40 (–1.00, 0.20)		–0.81 (–1.42, –0.20)*		–1.28 (–1.96, –0.61)**		–0.12 (–0.17, –0.06)		< 0.01
Birth (cm)		4,448		0.04 (–0.05, 0.13)		0.02 (–0.07, 0.12)		0.00 (–0.10, 0.11)		0.00 (–0.01, 0.01)		0.85
Length												
First trimester (mm)		1,541		–0.10 (–1.11, 0.91)		0.54 (–0.50, 1.57)		0.06 (–1.08, 1.20)		0.01 (–0.07, 0.08)		0.87
Second trimester (mm)		6,646		–0.08 (–0.20, 0.05)		–0.18 (–0.30, –0.05)*		–0.19 (–0.33, –0.06)*		–0.02 (–0.03, –0.01)		< 0.01
Third trimester (mm)		6,778		–0.02 (–0.17, 0.13)		–0.09 (–0.24, 0.06)		–0.33 (–0.50, –0.16)**		–0.02 (–0.04, –0.01)		< 0.01
Birth (cm)		5,606		–0.10 (–0.25, 0.05)		–0.01 (–0.17, 0.14)		–0.09 (–0.26, 0.09)		–0.01 (–0.02, 0.01)		0.49
Weight												
Second trimester (g)		6,612		–0.3 (–3.3, 2.6)		–1.4 (–4.4, 1.6)		0.8 (–2.5, 4.1)		0.1 (–0.2, 0.3)		0.67
Third trimester (g)		6,751		–3.7 (–15.8, 8.5)		–7.2 (–19.6, 5.1)		–14.2 (–28.0, –0.5*		–0.7 (–1.8, 0.5)		0.25
Birth (g)		7,003		2.6 (–25.0, 30.2)		–18.6 (–46.7, 9.6)		–37.6 (–69.7, –5.6)*		–3.4 (–6.2, –0.6)		0.02
Values are regression coefficients and reflect the difference in fetal growth parameters for each quartile of NO_2_ exposure (averaged from conception until measurement) compared with the reference group (lowest quartile). Cutoff values for categorization of NO_2_ exposure are < 37.0, 37.0–40.9, 40.9–43.9, > 43.9 μg/m^3^ for first trimester, < 37.0, 37.0–40.5, 40.5–43.4, > 43.4 μg/m^3^ for second trimester, < 37.0, 37.0–39.8, 39.8–42.8, > 42.8 μg/m^3^ for third trimester, and < 37.2, 37.2–39.6, 39.6–42.2, > 42.2 μg/m^3^ for total pregnancy. Models are adjusted for gestational age and noise exposure at measurement, maternal age, body mass index, height, parity, ethnicity, education, folic acid supplementation use, smoking, alcohol consumption, paternal height, and fetal sex. Models with neonatal head circumference or length are additionally adjusted for postconceptional age (gestational age for measurements at birth or gestational age plus postnatal age for measurements at the child health center), and models with neonatal length are additionally adjusted for method of measurement. *p < 0.05. **p < 0.001.												

When comparing the individual associations of maternal air pollution exposure and smoking during pregnancy with weight by trimester, we observed that inverse associations for air pollution exposure were smaller in magnitude than associations for maternal smoking but still considerable: Smoking compared with no smoking was associated with decreases of 39 g and 146 g in third-trimester weight and birth weight, respectively, whereas elevated PM_10_ and NO_2_ exposure levels (highest vs. lowest quartile) were associated with reductions of 13 g and 46 g in third-trimester weight and birth weight, respectively, for PM_10_ and reductions of 20 g and 61 g in third-trimester weight and birth weight, respectively, for NO_2_ (results not shown). When including both PM_10_ and NO_2_ in the models, the inverse association for PM_10_ exposure with first-trimester crown–rump length reached statistical significance, and the associations for PM_10_ exposure with third-trimester head circumference and for NO_2_ exposure with femur length in the second and third trimester persisted (results not shown). The unadjusted associations were consistent with the adjusted associations, although somewhat stronger inverse associations and smaller *p*-values were observed for PM_10_ exposure with weight in the third trimester and at birth, and for NO_2_ exposure with head circumference in the second and third trimester, length neonatally, and weight in the third trimester and at birth (results not shown).

**Figure 1:**
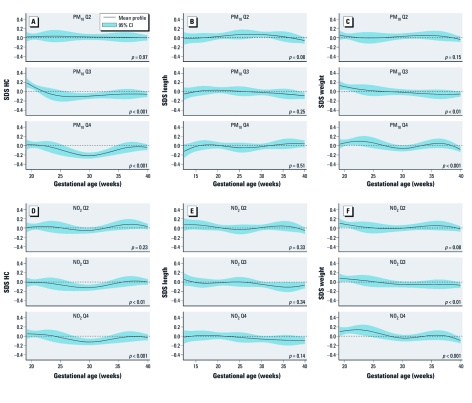
Associations of PM_10_ and NO_2_ exposure with longitudinally measured fetal growth characteristics. Figures are based on spline regression models of longitudinally measured (*A,D*) head circumference (HC) growth, 17,726 observations; (*B,E*) length growth, *n* = 20,305 observations; and (*C,F*) weight growth, *n* = 20,395 observations, all expressed in SD scores (SDS), for each quartile (Q) of air pollution exposure during pregnancy (from conception until delivery) compared with the reference group (lowest quartile). *A–C* present associations for PM_10_ exposure, and *D–F* present associations for NO_2_ exposure. Head circumference was estimated by ultrasound in second and third trimester of pregnancy and measured neonatally. Fetal length was measured by ultrasound as crown–rump length in first trimester and femur length in second and third trimester and as body length neonatally. Weight was estimated by ultrasound in second and third trimester of pregnancy and measured at birth. *p*-Values are based on multivariate *F*-tests and reflect the difference between the spline of each air pollution exposure category compared with the reference category. Models are adjusted for maternal age, body mass index, height, parity, ethnicity, education, folic acid supplementation use, smoking, alcohol consumption, noise exposure, paternal height, and fetal sex.

Figure 1 presents the associations of PM_10_ and NO_2_ exposure with longitudinally measured fetal growth. Compared with the first quartile, the third and fourth quartiles of PM_10_ and NO_2_ exposure showed a significant overall difference in head circumference growth during pregnancy (*p*-values < 0.01) (Figure 1A,D). No significant associations were observed for air pollution exposure with longitudinally measured fetal length (Figure 1B,E). Figure 1C,F shows significant overall differences in weight growth during pregnancy for the highest quartiles of PM_10_ and NO_2_ exposure compared with the first quartile (*p* < 0.01 and *p* < 0.001 for third and fourth quartiles, respectively).

*Air pollution and risks of adverse birth outcomes.* Compared with mothers in the lowest quartile of PM_10_ exposure levels, exposures in the third and fourth quartiles were positively associated with preterm birth [odds ratio (OR) = 1.40, 95% CI: 1.03, 1.89; and OR = 1.32, 95% CI: 0.96, 1.79, respectively] (Table 5). The third quartile of PM_10_ exposure was associated with SGA (OR = 1.38; 95% CI: 1.00, 1.90), but no significant association was observed for the fourth quartile (OR = 1.23; 95% CI: 0.89, 1.70). No consistent associations were observed for NO_2_ exposure with adverse birth outcomes. When including both PM_10_ and NO_2_ in the models, associations for PM_10_ exposure with preterm birth became larger in magnitude, and larger effect estimates with smaller *p*-values were observed for associations between PM_10_ exposure and low birth weight (results not shown). Relative to the adjusted models, the unadjusted models showed smaller *p*-values for the positive associations for PM_10_ exposure with preterm birth and SGA at birth (*p*-values for trend = 0.03) and larger effect estimates with smaller *p*-values for the associations for NO_2_ exposure with preterm birth, low birth weight, and SGA at birth (e.g., for SGA at birth: OR = 1.47, 95% CI: 1.08, 2.01; and OR = 1.49, 95% CI: 1.09, 2.04 for the third and fourth quartiles of NO_2_ exposure, respectively) (results not shown).

**Table 5:** Associations of PM_10_ and NO_2_ exposure with the risks of adverse birth outcomes [OR (95% CI)].

Air pollution exposure	*n*	Preterm birth (< 37 weeks) (*n *= 7,045)	*n*	Low birth weight (< 2,500 g) (*n* = 7,003)	*n*	SGA at birth (< 5%) (*n* = 6,997)
PM_10_												
First quartile		78		Reference		74		Reference		73		Reference
Second quartile		75		0.96 (0.70, 1.33)		66		0.76 (0.49, 1.20)		78		1.05 (0.75, 1.47)
Third quartile		106		1.40 (1.03, 1.89)*		93		0.89 (0.58, 1.34)		98		1.38 (1.00, 1.90)*
Fourth quartile		105		1.32 (0.96, 1.79)^#^		90		0.91 (0.60, 1.40)		97		1.23 (0.89, 1.70)
Trend test (per 1-µg/m^3^ increase)				1.03 (1.00, 1.07)				1.00 (0.95, 1.05)				1.03 (0.99, 1.07)
*p*-Value for trend				0.07				0.93				0.13
NO_2_												
First quartile		79		Reference		75		Reference		70		Reference
Second quartile		92		1.10 (0.81, 1.51)		71		0.84 (0.54, 1.31)		73		0.93 (0.66, 1.31)
Third quartile		95		1.09 (0.79, 1.49)		88		0.86 (0.55, 1.33)		101		1.25 (0.90, 1.73)
Fourth quartile		99		1.10 (0.77, 1.57)		89		0.95 (0.58, 1.55)		102		1.35 (0.94, 1.94)
Trend test (per 1-µg/m^3^ increase)				1.01 (0.98, 1.04)				1.00 (0.95, 1.04)				1.03 (0.99, 1.06)
*p*-Value for trend				0.43				0.87				0.11
Values are ORs (95% CI) and reflect the risk for adverse birth outcomes for each quartile of air pollution exposure during pregnancy (from conception until delivery) compared with the reference group (lowest quartile). Cutoff values for categorization are < 27.8, 27.8–30.0, 30.0–32.9, > 32.9 μg/m^3^ for PM_10_ exposure and < 37.2, 37.2–39.6, 39.6–42.2, > 42.2 μg/m^3^ for NO_2_ exposure. Models are adjusted for maternal age, body mass index, height, parity, ethnicity, education, folic acid supplementation use, smoking, alcohol consumption, noise exposure, and paternal height. Models with preterm birth and low birth weight are additionally adjusted for fetal sex, and models with low birth weight are additionally adjusted for gestational age at birth. *p < 0.05. ^#^*p* < 0.10.												

## Discussion

Results from this large population-based prospective cohort study from early pregnancy onward suggest that maternal exposure to PM_10_ and NO_2_ is inversely associated with fetal growth in the second and third trimester and with weight at birth. Elevated PM_10_ exposure levels were also positively associated with preterm birth and SGA.

*Air pollution, fetal growth, and birth outcomes.* A few animal experiments suggested effects of maternal exposure to air pollution on placental function and fetal growth (Rocha et al. 2008; Tsukue et al. 2002). Several potential biological mechanisms have been described through which air pollution could influence pregnancy, such as induction of systemic inflammation and oxidative stress (Kannan et al. 2006), eventually resulting in suboptimal placentation (Dejmek et al. 1999) and increased maternal susceptibility to infections (Slama et al. 2008a). These alterations could impair fetal growth and result in adverse birth outcomes.

Thus far, only three studies have examined associations of maternal air pollution exposure with fetal growth measured by ultrasound during pregnancy. The first study was conducted in Australia among 14,734 women and examined 15,623 midpregnancy ultrasound scans. The researchers observed inverse associations of maternal exposure to PM_10_, ozone (O_3_), and sulfur dioxide (SO_2_) during different periods with fetal growth parameters. No significant associations were observed for NO_2_ exposure. The authors reported that the observed associations were heterogeneous regarding the specific exposure periods and outcome measures examined (Hansen et al. 2008). The second study was conducted in France based on three ultrasound scans in 271 women. Associations were observed between maternal personal exposure to airborne benzene and smaller fetal biparietal diameter in mid- and late pregnancy and with head circumference in mid- and late pregnancy and at birth (Slama et al. 2009). The third study was conducted in Spain among 562 pregnant women with 1,692 scans and observed no associations for NO_2_ exposure with fetal growth parameters in different periods. When the analysis was restricted to women who spent < 2 hr/day in nonresidential outdoor environments, significant associations were observed between exposure to a mixture of aromatic hydrocarbons (benzene, toluene, ethylbenzene, and xylene; BTEX) and biparietal diameter growth during the second trimester and between NO_2_ exposure and SD scores for both size and growth of second- and third-trimester head circumference, abdominal circumference, biparietal diameter, and estimated fetal weight (Aguilera et al. 2010).

Several studies have estimated the impact of air pollution on anthropometric parameters at birth such as head circumference, length, and weight. Inverse associations of maternal exposure to NO_2_ (Ballester et al. 2010), polycyclic aromatic hydrocarbons (PAHs) (Choi et al. 2006), and particulate matter with an aerodynamic diameter < 2.5 μm (PM_2.5_) (Jedrychowski et al. 2004) with head circumference and length at birth have been reported. A reduction in birth weight has also been linked to air pollutants, including PM_2.5_ (Jedrychowski et al. 2004), PAHs (Choi et al. 2006), and carbon monoxide (CO) and O_3_ (Salam et al. 2005). Another study has not detected associations between exposure to NO_2_, PM_10_, O_3,_ or visibility-reducing particles with head circumference and weight at birth (Hansen et al. 2007).

The present study was based on a larger number of fetal ultrasound measurements than previous studies. We observed an inconsistent pattern of associations for air pollution across gestation, which was reported earlier as well (Hansen et al. 2008). The clinical relevance of a relative decrease in head and length growth during pregnancy when sizes at birth are within the normal range needs to be further studied, as well as the consequences of a relative increase in weight during pregnancy followed by a relative decrease in weight at birth. However, results from the analyses at different time points should be interpreted carefully, because the number of subjects with available outcome data, and hence the statistical power of the analyses, varied between measurements in our study. Also, differences in methods and accuracy between fetal and neonatal measurements could explain the heterogeneous results. We estimated small differences in fetal growth parameters. For example, in the third trimester, the highest PM_10_ and NO_2_ exposure quartiles were associated with a reduction in femur length of 0.2 and 0.3 mm and a reduction in head circumference of 1.7 and 1.3 mm, respectively. These differences may not be clinically relevant on an individual level but could be relevant on a population level. Moreover, although we have previously shown good intra- and interobserver reproducibility of fetal biometry measurements (Verburg et al. 2008a), the associations might be underestimated because of random measurement error. Although the overall strength of evidence is still limited, the results of previous studies and our study suggest that air pollution exposure influences fetal growth from the second trimester onward. We observed associations for PM_10_ exposure, but not NO_2_ exposure, with preterm birth and SGA. The literature on birth outcomes has increased in the last decade, which has led to a number of reviews summarizing the available evidence (Bonzini et al. 2010; Glinianaia et al. 2004; Lacasaña et al. 2005; Maisonet et al. 2004; Shah and Balkhair et al. 2011; Šrám et al. 2005). Most routinely measured air pollutants (e.g., PM_10_, PM_2.5_, NO_2_, CO, O_3_, SO_2_) have been linked to outcomes such as preterm birth, low birth weight, and intrauterine growth restriction (Ritz and Wilhelm 2008), but results differ among studies. In our previous work, residential proximity to traffic—a proxy for traffic-related air pollution—was not consistently associated with birth weight nor with preterm birth and SGA (van den Hooven et al. 2009). In this study, we were able to estimate individual exposure levels that better capture the spatial and temporal variation in air pollution concentrations.

Air pollution, especially the traffic-related part, is a complex mixture of several pollutants. PM_10_ and NO_2_ might act as surrogates for this mixture and are therefore not necessarily the causative agents in the relation between air pollution and adverse fetal growth and birth outcomes. The biological plausibility of health effects induced by particulate matter has been well described (Kannan et al. 2006; Slama et al. 2008a). In contrast, it has been proposed that health risks associated with NO_2_ may result from traffic-related emissions correlating with NO_2_, chemical reaction products of NO_2_, or NO_2_ itself (World Health Organization 2006). When including both PM_10_ and NO_2_ in the models, the results did not highlight clearly stronger associations for one pollutant or the other. We acknowledge that the variation in exposure levels is relatively small in our study population. In populations with a larger exposure variability, stronger associations for air pollution exposure with fetal growth parameters and adverse birth outcomes might be detected.

*Methodological considerations.* Many previous studies assessed exposure to air pollution using only monitoring stations. That approach does not consider intra-urban gradients in pollutants. More recent approaches applying spatial modeling address the spatial variation but not the temporal variation. Together with a number of recent studies that used temporally adjusted land-use regression models or dispersion models to assess exposure (Aguilera et al. 2010; Ballester et al. 2010; Gehring et al. 2011; Slama et al. 2009), we were able to consider finer spatial and temporal contrasts in exposure by using a combination of dispersion modeling and continuous monitoring. The quality of the assigned exposure estimates was further enhanced by allowing for residential mobility of the women during pregnancy, which overcomes the potential misclassification that could arise when exposure is based solely on the home address at time of delivery (Fell et al. 2004). There might still be nondifferential misclassification of air pollution exposure. Exposure levels were estimated at the home address; however, pregnant women do not spend all of their time at home. Other types of exposure (e.g., occupational or commuting) were not taken into account.

Although fetal ultrasound examination is a more reliable basis than the LMP for establishing gestational age (Tunon et al. 1996), this method has the disadvantage that the growth variation of the fetal characteristics used for pregnancy dating is assumed to be zero (Verburg et al. 2008b). Because the early pregnancy characteristics are correlated throughout pregnancy with head circumference, abdominal circumference, and femur length, our study may have underestimated the variation in the latter three growth characteristics, resulting in an underestimation of our effect estimates. In addition, the assessment of gestational age could be biased if air pollution exposure shows an early effect of fetal growth (Slama et al. 2008a). We observed a nonsignificant inverse association between PM_10_ exposure and crown–rump length. When restricting the analyses to the subgroup of women with a known LMP, adjustment for the LMP-based gestational age rather than the ultrasound-based gestational age resulted in somewhat stronger negative effects of air pollution on fetal growth from the third trimester onward. This suggests that effects of air pollution on fetal growth might be underestimated when gestational age is defined using ultrasounds (Slama et al. 2008b). However, the observations in this subgroup should be considered with caution because of the relatively small size.

## Conclusions

This prospective population-based cohort study in the Netherlands suggests that maternal PM_10_ and NO_2_ exposure is inversely associated with fetal growth during the second and third trimester and with weight at birth. Elevated PM_10_ exposure was also associated with preterm birth and SGA. This study further supports previous epidemiologic research and suggests that the associations between maternal exposure to air pollution and fetal growth are trimester- and growth characteristic–specific. Future studies are needed to explore the underlying mechanisms and postnatal consequences of these findings.

## Supplemental Material

(881 KB) PDF
