# Supplementary material for: Air Pollution Exposure During Pregnancy, Ultrasound Measures of Fetal Growth, and Adverse Birth Outcomes: A Prospective Cohort Study
Source: Environ Health Perspect. 2011 Aug 26;120(1):150–6. doi: 10.1289/ehp.1003316 (PMC3261932; doi:10.1289/ehp.1003316)
Supplement: (881 KB) PDF [file ehp.1003316.s001.pdf]

Supplemental Material

Air Pollution Exposure During Pregnancy, Ultrasound Measures of Fetal Growth, and Adverse Birth Outcomes: A Prospective Cohort Study

Edith H. van den Hooven, Frank H. Pierik, Yvonne de Kluizenaar, Sten P. Willemsen, Albert Hofman, Sjoerd W. van Ratingen, Peter Y.J. Zandveld, Johan P. Mackenbach, Eric A.P. Steegers, Henk M.E. Miedema, Vincent W.V. Jaddoe

Table of Contents

2 Figure S1. Population for analysis

3 File S1. Air pollution exposure assessment

7 Figure S2. Flow chart of air pollution exposure assessment

8 File S2. Traffic-related noise exposure assessment

10 Figure S3. Maps of the study area showing annual average PM_{10} and NO_2 concentrations in 2003 at the home addresses

12 References (Supplemental Material)

Supplemental Material, Figure S1. Population for analysis.

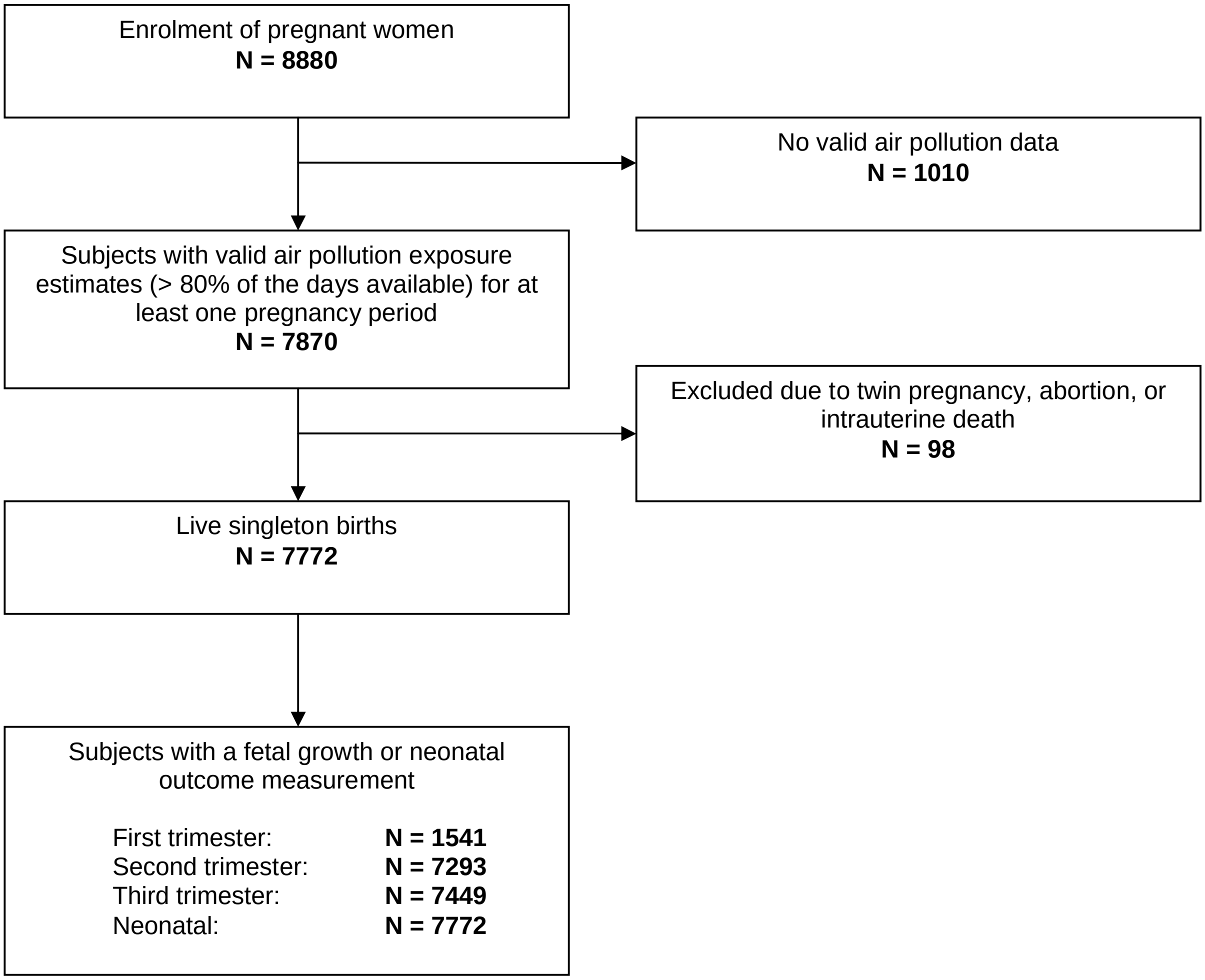

Supplemental Material, File S1. Air pollution exposure assessment

Hourly concentrations of particulate matter (PM_{10}) and nitrogen dioxide (NO_2) at all addresses in the study area (Northern part of Rotterdam) were estimated for the years 2001-2006, using advanced spatiotemporal dispersion modelling techniques in combination with hourly air pollution measurements at three continuous monitoring sites located around the study area. A flow chart of the exposure assessment approach is presented in Figure S2. We took the following steps to assign exposure estimates to the addresses in this area.

Spatial pattern

First, approximately 800 000 digital calculation points, further referred to as ‘receptors’, were assigned to the façades of all dwellings. Annual average concentrations of PM_{10} and NO_2 were assessed for every receptor using the three Dutch national standard methods for the calculation of air quality, and Geographic Information Systems (GIS). These standard methods have been established by the Dutch government, and are designated to calculate the contribution of intra-urban road traffic, traffic on highways, and industrial and other point sources (standard calculation method 1, 2 and 3, respectively) (VROM (Netherlands Ministry of Housing Spatial Planning and the Environment) 2007). For each year, the spatial distribution of annual average concentrations was assessed for eight different wind conditions (wind direction: north/east/south/west; wind velocity: light/strong). For each hour, we then derived the corresponding spatial distribution for the prevailing wind direction and wind speed at that specific hour, by means of interpolation between the eight characteristic spatial distributions.

Input data for the calculations described above were traffic characteristics (including traffic intensities, traffic composition, and traffic speed), road characteristics, vehicle emission factors, buildings and ground characteristics, and emission data from shipping, industry, and households. Detailed digital maps with information on geographic locations and traffic characteristics for roads in the study area were obtained from the local authorities of Rotterdam. Traffic intensities and meteorological data were supplied by the DCMR Environmental Protection Agency Rijnmond (DCMR).

Emission sources and emission data were obtained from the National Institute for Public Health and the Environment (RIVM) and the DCMR.

Subsequently, the spatial distributions that corresponded to the hourly wind conditions were adjusted for fixed temporal patterns of source activities, by applying fixed scaling factors to the contributions of various air pollution sources. In this way, we accounted for temporal fluctuations in the contribution of air pollution sources during the day (e.g., morning and evening rush hour), week (e.g., working days and weekend days), and month. For example, the contribution of traffic was scaled with the hourly traffic intensity pattern. The fixed temporal patterns were derived on the basis of traffic counts (reflecting traffic fluctuation patterns) and energy usage data (reflecting residential heating patterns).

Temporal pattern and calibration

The modelled concentrations were adjusted based on hourly continuous monitoring data at three stations in the area. This served two main purposes. First, the temporal fluctuations in background concentrations were taken into account. Second, the modelled concentrations were calibrated against measured concentrations.

The hourly calibration procedure was performed in the following way. Concentrations were modelled for each hour at the locations of three monitoring stations and compared with the actual measurements. Subsequently, the differences between modelled and measured concentrations at the three monitoring stations were averaged into a representative difference for the whole area. This difference was added to or subtracted from the modelled concentrations.

Continuous air pollution monitoring data was provided by DCMR. Missing values for PM_{10} concentrations at the three monitoring stations were imputed with hourly concentrations derived from the large-scale concentration database for air pollution in the Netherlands (generally referred to as ‘GCN map’) published by the Netherlands Environmental Assessment Agency (PBL), the national institute for policy analysis in the field of environment, nature and spatial planning. The hourly concentrations in the GCN database are estimated on the basis of hourly measurements from the National Air Quality Monitoring Network (LML), emission data, and modelling. The developed nationwide concentration

maps are updated annually and provide a best estimate of large-scale air quality currently available (Velders et al. 2010).

Modelling performance

Two recent studies examined the performance of a dispersion modelling approach that incorporates the same Dutch national standard models for calculation of air pollution concentrations in the larger Rotterdam region. These studies showed a good agreement between annual average NO_2 concentrations predicted by dispersion modelling and concentrations measured at sixteen sites in the area (Pearson correlation coefficient ρ=0.77) (Beelen et al. 2010) and a good agreement between predicted annual average PM_{10} concentrations and concentrations measured at three sites in the area (Keuken et al. 2011). It can be expected that the hourly PM_{10} and NO_2 concentrations estimated for the present study will be more precise than annual average concentrations, since the substantial temporal fluctuations in air pollution concentrations were taken into account by the hourly calibration procedure with hourly monitoring data, which further enhances the agreement between predicted and measured concentrations.

Exposure assignment

For each dwelling, the receptor at the most exposed façade was selected, and the corresponding air pollution values were assigned to the address. We obtained full residential history of the participants by combining the address data collected by questionnaires with data from the local authorities of Rotterdam. It was ascertained that the residential history covered the total pregnancy period. Of the women in our study, 87% did not move during pregnancy, 12% changed residence once, 1% moved twice, and 0.1% moved three times during pregnancy.

We calculated exposure estimates for the participants using the following approach. Derived from the hourly concentrations of PM_{10} and NO_2, we constructed a database containing daily averages (24h) for every address, for the years 2001-2006. Allowing for residential mobility, air pollution exposure estimates were linked to the different home addresses of the participants during pregnancy. We derived average exposure estimates for different periods in pregnancy: 1) conception until first trimester ultrasound (median 13.2 weeks of gestation, 95% range 10.5 to 17.5); 2) conception until second

trimester ultrasound (median 20.5 weeks of gestation, 95% range 18.6 to 23.4); 3) conception until early third trimester ultrasound (median 30.3 weeks of gestation, 95% range 28.3 to 33.0); and 4) conception until delivery (median 40.1 weeks of gestation, 95% range 35.5 to 42.4). Average exposures were only calculated for periods with <20% of the daily averages missing. For the other periods, air pollution exposures were set to missing. There was substantial spatial and temporal variation in pregnancy-specific exposure levels. Distributions of PM_{10} and NO_2 exposure levels in our study population are presented in Table 2.

Supplemental Material, Figure S2. Flow chart of the air pollution exposure assessment.

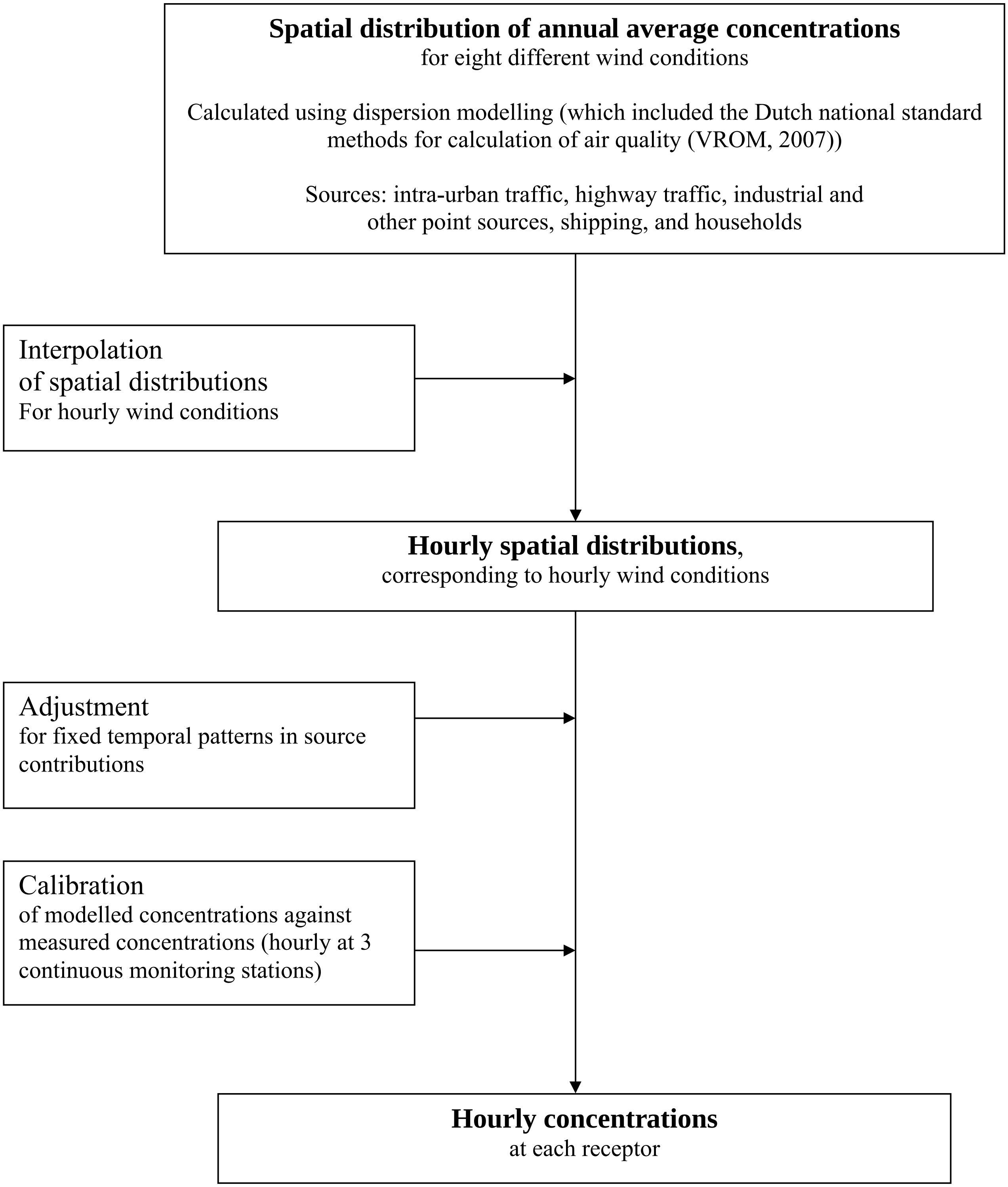

Supplemental Material, File S2. Traffic-related noise exposure assessment

We adjusted for noise exposure when examining the associations for air pollution exposure with fetal growth and neonatal complications. Recent studies expressed the need to include information on noise exposure in studies on traffic-related air pollution exposure and health, since traffic is a major shared source for both air pollution and noise (Allen et al. 2009; Davies et al. 2009; de Kluizenaar et al. 2007; de Kluizenaar et al. 2009). Noise is hypothesized to induce stress responses, which may result in altered function of the sympathetic autonomic nervous system, endocrine system, and immune system (Babisch 2000; Passchier-Vermeer and Passchier 2000), possibly contributing to an increased risk for an unfavorable pregnancy outcome (Magann et al. 2005; Nurminen 1995; Passchier-Vermeer and Passchier 2000).

Road traffic noise exposure was assessed in accordance with requirements of the European Environmental Noise Directive. The method has been described previously in more detail (de Kluizenaar et al. 2007; de Kluizenaar et al. 2009). Input data for the noise calculations was a detailed digital map describing the geographic location of roads and the traffic characteristics for each road segment, provided by the local authorities of Rotterdam for the current situation at time of the study (base year 2004). This data can be reasonably applied to adjacent years, as the road network is assumed to be rather stable, with only small (if any) but equal changes in noise exposure across the population. Noise exposure levels are expressed in the EU standard noise metric L_{den} (day, evening, night), a measure of annual average sound levels. We assessed the road traffic noise (L_{den}) level at the most exposed façade of the dwelling of every address in the study area. Very low levels of noise exposure (<45 dB(A)) were recoded as 45 dB(A) because this was considered a lower limit of the ambient noise in urban surroundings. To each participant, we assigned the noise exposure level calculated at the participant's home address at time of the ultrasound or neonatal outcome measurement. Mean noise exposure was 52.7 (95% range 45.0 to 68.3) dB(A) at time of the first trimester ultrasound; 52.8 (95% range 45.0 to 68.2) dB(A) at time of the second trimester ultrasound; 52.7 (95% range 45.0 to 68.2) dB(A) at time of the third trimester ultrasound; and 52.7 (95% range 45.0 to 68.2) db(A) at delivery.

Similar to air pollution exposure, noise exposure was calculated with a detailed model that takes into account the small scale intra-urban spatial contrasts in the study area. This approach reduces the misclassification of noise exposure that may occur in studies where exposure is based for example on calculations for a coarse grid or on subjective information such as questionnaire data. Furthermore, both air pollution exposure and noise exposure were assessed using the same spatial input data, and for the same locations (the façades of participants' home addresses), thereby reducing potential bias resulting from differences in spatial resolution.

Supplemental Material, Figure S3. Maps of the study area showing annual average PM_{10} and NO_2 concentrations in 2003 at the home addresses.

a. PM_{10} concentration

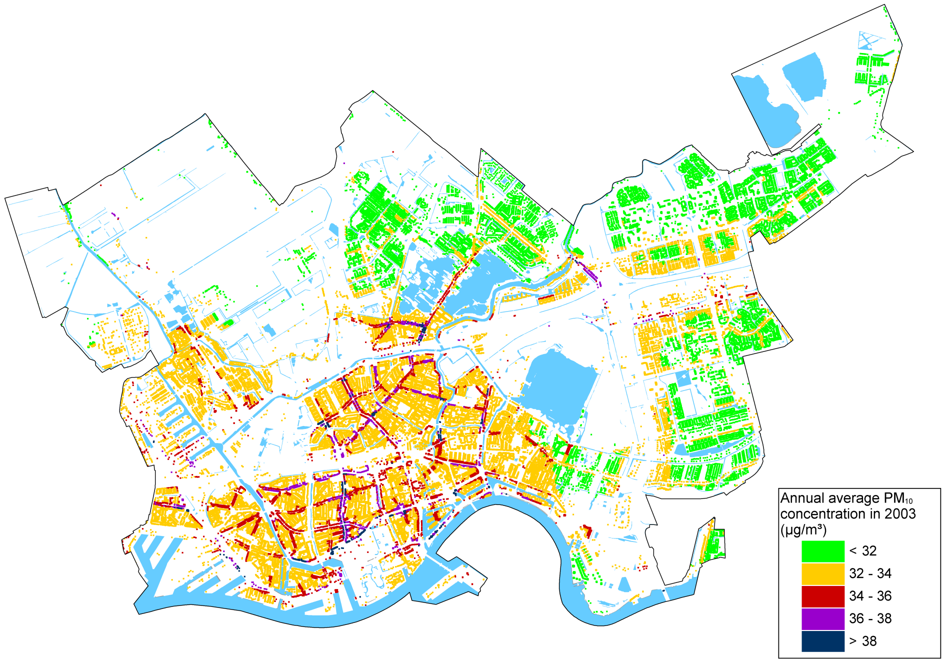

b. NO_2 concentration

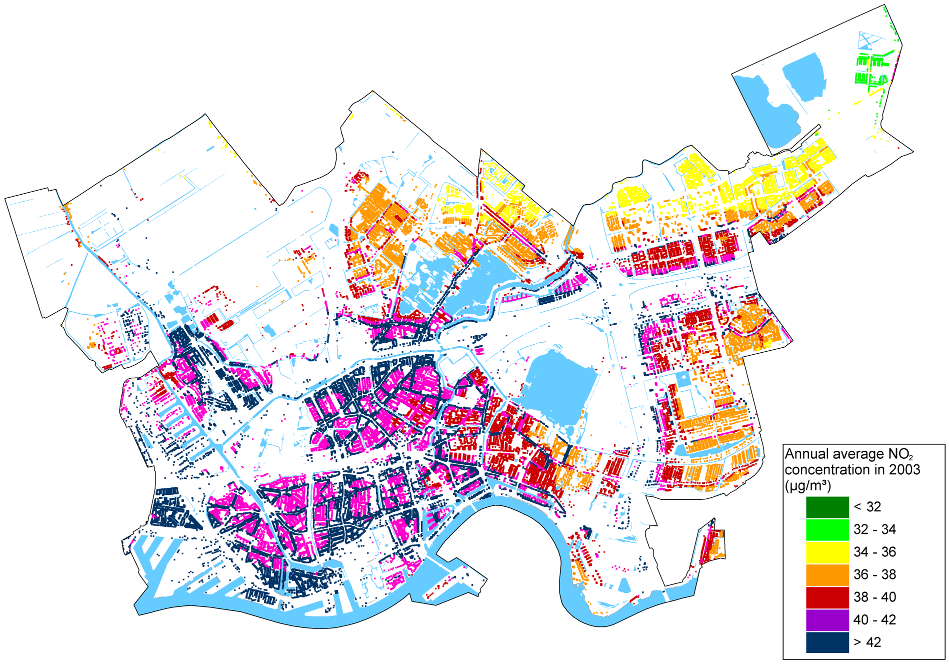

The maps show the surface water (in blue) and the spatial distribution of PM_{10} and NO_2 concentrations (see legends) in the study area. Areas without addresses remain white on the maps. The presented PM_{10} and NO_2 concentrations are annual average concentrations in 2003 for the addresses in the study area. These figures are only presented as an illustration, since daily average concentrations in 2001-2006 were used to calculate individual exposures.

REFERENCES (Supplemental Material)

Allen RW, Davies H, Cohen MA, Mallach G, Kaufman JD, Adar SD. 2009. The spatial relationship between traffic-generated air pollution and noise in 2 US cities. Environ Res 109:334-342.

Babisch W. 2000. Traffic Noise and Cardiovascular Disease: Epidemiological Review and Synthesis. Noise Health 2:9-32.

Beelen R, Voogt M, Duyzer J, Zandveld P, Hoek G. 2010. Comparison of the performances of land use regression modelling and dispersion modelling in estimating small-scale variations in long-term air pollution concentrations in a Dutch urban area. Atmospheric Environment 44:4614-4621.

Davies HW, Vlaanderen JJ, Henderson SB, Brauer M. 2009. Correlation between co-exposures to noise and air pollution from traffic sources. Occup Environ Med 66:347-350.

de Kluizenaar Y, Gansevoort RT, Miedema HM, de Jong PE. 2007. Hypertension and road traffic noise exposure. J Occup Environ Med 49:484-492.

de Kluizenaar Y, Janssen SA, van Lenthe FJ, Miedema HM, Mackenbach JP. 2009. Long-term road traffic noise exposure is associated with an increase in morning tiredness. J Acoust Soc Am 126:626-633.

Keuken M, Zandveld P, van den Elshout S, Janssen NAH, Hoek G. 2011. Air quality and health impact of PM10 and EC in the city of Rotterdam, the Netherlands in 1985-2008. Atmospheric Environment 45:5294-5301.

Magann EF, Evans SF, Chauhan SP, Nolan TE, Henderson J, Klausen JH, et al. 2005. The effects of standing, lifting and noise exposure on preterm birth, growth restriction, and perinatal death in healthy low-risk working military women. J Matern Fetal Neonatal Med 18:155-162.

Nurminen T. 1995. Female noise exposure, shift work, and reproduction. J Occup Environ Med 37:945-950.

Passchier-Vermeer W, Passchier WF. 2000. Noise exposure and public health. Environ Health Perspect 108 Suppl 1:123-131.

Velders G, Aben J, Diederen H, Drissen E, Geilenkirchen G, Jimmink B, et al. 2010. Large-scale Air Quality Concentration Maps in the Netherlands, Report 2010 (Concentratiekaarten voor grootschalige luchtverontreiniging in Nederland, Rapportage 2010). PBL Report 500088006. Available: http://www.rivm.nl/bibliotheek/rapporten/500088006.pdf.

VROM (Netherlands Ministry of Housing Spatial Planning and the Environment). 2007. Air Quality Decree (Regeling beoordeling Luchtkwaliteit 2007).
